# Dephasing rates for weak localization and universal conductance fluctuations in two dimensional Si:P and Ge:P *δ*-layers

**DOI:** 10.1038/srep46670

**Published:** 2017-05-04

**Authors:** Saquib Shamim, S. Mahapatra, G. Scappucci, W. M. Klesse, M. Y. Simmons, Arindam Ghosh

**Affiliations:** 1 Department of Physics, Indian Institute of Science, Bangalore 560 012, India; 2 Centre for Quantum Computation and Communication Technology, University of New South Wales, Sydney NSW 2052, Australia

## Abstract

We report quantum transport measurements on two dimensional (2D) Si:P and Ge:P *δ*-layers and compare the inelastic scattering rates relevant for weak localization (WL) and universal conductance fluctuations (UCF) for devices of various doping densities (0.3–2.5 × 10^18^ m^−2^) at low temperatures (0.3–4.2 K). The phase breaking rate extracted experimentally from measurements of WL correction to conductivity and UCF agree well with each other within the entire temperature range. This establishes that WL and UCF, being the outcome of quantum interference phenomena, are governed by the same dephasing rate.

Delta doped Si:P and Ge:P devices offer an excellent platform for devices in quantum circuits due to their exceptionally low electrical noise^1^. Experimental and theoretical investigations of *δ*-doped devices over the past decade have led to realization of high conductivity interconnects with extremely low noise^2^ and single/few donor based devices of phosphorous in silicon^3^. These delta-doped devices have been made possible through scanning tunneling microscope (STM) lithography combined with molecular beam epitaxy (MBE). This technological advancement in STM-MBE fabrication takes us forward to address the solid state quantum bits and perform the required quantum operation. These systems, though aimed to be the building blocks of a solid state quantum computation scheme can have exotic and novel physical properties as they are a natural realization of a half-filled impurity band in 2D. The P *δ*-layers in Si and Ge provide a pure semiconductor based two dimensional (2D) electron system to investigate quantum coherent phenomena such as weak localization (WL) and universal conductance fluctuations (UCF). Here, we compare the phase breaking rate (which is the inverse of phase coherence time) relevant to the phenomena of WL, 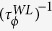, and UCF, 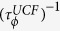, in *δ*-doped 2D electron systems in silicon and germanium. Using measurements of magnetoconductance and time-dependent conductance fluctuations in a perpendicular magnetic field, we show that 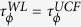 which implies that WL and UCF are governed by the same scattering rates.

Quantum transport in low dimensional disordered systems at low temperatures is highly sensitive to the phase coherence of interfering wave functions of electrons. Investigation of quantum transport in disordered systems have often been carried out by measuring the temperature dependence of conductivity, the magnetoconductivity in a perpendicular magnetic field 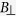 or through conductivity fluctuations. The coherent interference of wave functions of electrons as they are multiply backscattered by random impurities lead to the ubiquitous phenomena of WL and UCF which are fundamental concepts in disordered systems and have been extensively studied both experimentally and theoretically for a variety of systems over the past few decades^4^,^5^,^6^,^7^,^8^,^9^,^10^,^11^,^12^,^13^,^14^,^15^,^16^. WL is characterized by a reduction in conductivity as the temperature is reduced^6^,^17^,^18^ and the effect can be destroyed by the application of 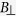 which changes the phase of the interfering waves. The UCF are aperiodic fluctuations in conductance *G* as a function of magnetic field, Fermi energy or disorder potential with a universal magnitude 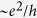 (Ref. 15). The fluctuations are most prominent when the sample dimensions are of the order of the phase coherence length 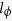 of the sample. Both WL and UCF are characteristic to the diffusive regime of electron transport where 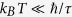, where *τ* is the elastic scattering time.

It is known that the time scale determining the WL correction and its temperature dependence is the phase breaking time 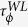, which can be experimentally evaluated by fitting the WL theory of disordered 2D conductors to the magnetoconductivity in 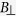
12,19,20,21,22. The relevant rate for UCF 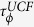 however, has been a subject of controversy with different theoretical and experimental reports over the last two decades^23^,^24^,^25^,^26^,^27^,^28^. The scattering time relevant to the expression for UCF was thought to be the out-scattering time 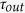 which is related to the frequency of inelastic collisions and differs from 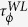 by a logarithmic factor for 2D systems^24^. This scenario was supported by experiments on quasi-2D thin silver films by Hoadley *et al*. who carried out WL and time-dependent UCF measurements to extract 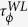 and 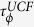 and found that 
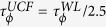
 for low temperatures where the electron-electron interactions cause dephasing^25^. This was contradicted by later theoretical work of Aleiner and Blanter who showed that the time scales for WL and UCF are exactly identical for dephasing by electron-electron scattering^26^. Specifically, Aleiner and Blanter showed that UCF is governed by the time-scale defined by a pole in the UCF diffuson which is exactly identical to the time scale for WL. Subsequent experiments by Trionfi *et al*. have supported this claim by demonstrating excellent agreement between 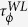 and 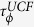 extracted from WL and UCF measurements respectively, for quasi-one dimensional (1D) and quasi-2D AuPd wires^27^. Thus there exists contradicting experimental and theoretical reports regarding the equivalence of 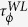 and 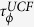. Trionfi *et al*. have suggested that the discrepancy between 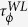 and 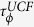 observed in some experiments, could result from an inaccurate expression for the magnetic field dependence of UCF (due to incorrect assumption that the UCFs are unsaturated)^28^.

In this report, we have studied the quantum transport in atomically thin P *δ*-layers in elemental semiconductors Si and Ge through the measurements of WL and UCF. We show that the phase breaking time relevant for WL and UCF are identical (i.e. 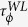 = 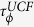) for the Si:P and Ge:P *δ*-layers at low temperatures (0.3–4.2 K) where the dephasing is due to electron-electron scattering as confirmed by the temperature dependence of 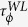. Our results are significant from the material aspect since *δ*-doped systems are the building blocks of solid state quantum computation architecture. From the fundamental physics aspect, we have studied the existing problem of the equivalence of 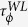 and 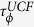 in atomically thin 2D systems as opposed to quasi-2D systems and thin films investigated earlier.

## Results

**Figure 1:**
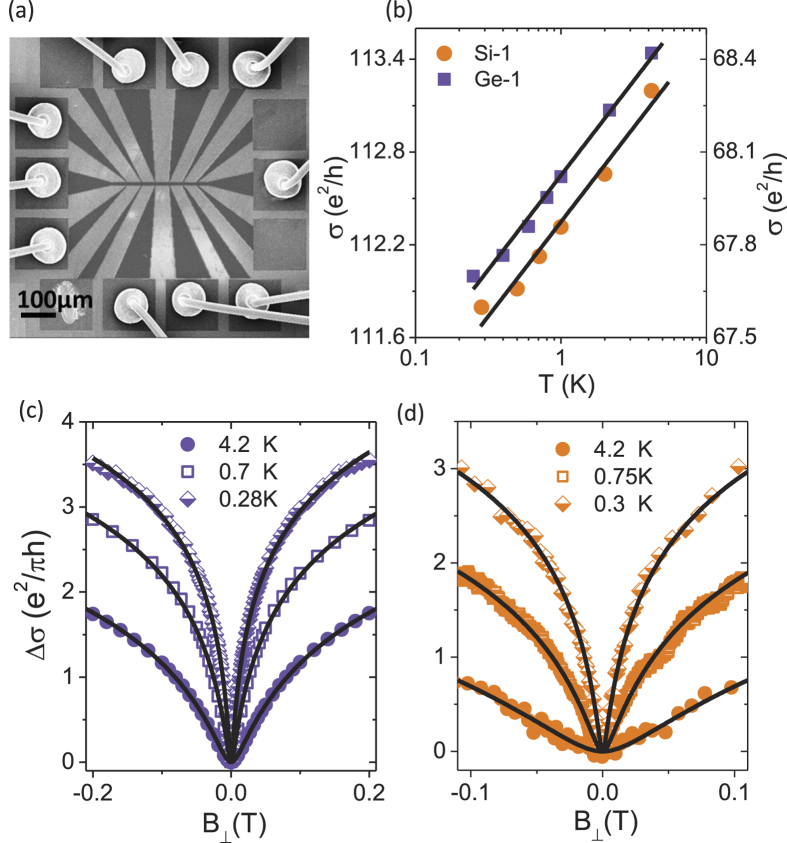
Quantum transport in Si:P and Ge:P *δ*-layers. (**a**) The scanning electron microscope image (false color) of a Ge:P *δ*-doped Hall bar used for measurements. (**b**) The temperature dependence of conductivity *σ* of heavily doped *δ*-layers Si-1 and Ge-1. The magnetoconductivity, 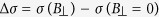, in a perpendicular magnetic field 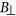 for (**c**) Ge:P *δ*-layer Ge-1 and (d) Si:P *δ*-layer Si-1 at different temperatures. The solid black lines are weak localization fits to magnetoconductivity (Eq. 1 of the main text).

All the Si:P and Ge:P *δ*-layers were fabricated in an ultra-high vacuum (UHV) variable-temperature STM system with a base pressure of 5 × 10^−11^ mbar. The STM-chamber has PH_3_ dosing system for P doping and a sublimation source (Si or Ge) for homoepitaxial growth. For electrical transport measurements, Hall bars were formed using electron beam lithography (EBL) and reactive ion etching. Ohmic contacts were made to the Hall bars by EBL and subsequent metallization. The details of the growth and fabrication process can be found in refs 29, 30, 31. A false color scanning electron microscope image of a Ge:P *δ*-doped Hall bar is shown in Fig. 1a.

### Quantum transport in Si:P and Ge:P *δ*-layers

The effect of quantum coherent transport in these devices is manifested in the logarithmic decrease of conductivity with temperature as shown in Fig. 1b for Si-1 and Ge-1. This logarithmic decrease in conductivity arises due to corrections from WL and electron-electron interaction effects as has been observed previously^32^. The characteristic feature of WL is a dip in the magnetoconductivity in a perpendicular magnetic field, 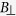, at 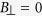 as shown in Fig. 1c and d for device Ge-1 and Si-1 respectively at different temperatures. To extract 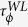, the magnetoconductivity 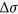 has been fitted with the Hikami formula for a disordered 2D system^12^,


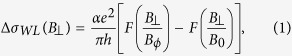


where 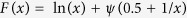, and 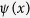 is the digamma function, 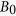 is the momentum relaxation field and 
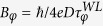
 is the phase breaking field with *D* being the electron diffusivity. 
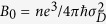
, has been estimated using the experimentally calculated Drude conductivity 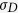 and number density *n* obtained from Hall measurements. The phenomenological prefactor *α* has a value in the range 1–1.2 for all devices at all temperatures. The solid black lines in Fig. 1c and d are the fits using Eq. 1 for the devices Ge-1 and Si-1 respectively with 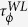 as the fitting parameter. Thus the phase breaking rate relevant to the phenomena of WL has been extracted for all devices at all temperatures and it agrees well with earlier reports on Si:P and Ge:P *δ*-layers^29^,^32^.

### Magneto-fingerprinting and noise due to UCF

**Figure 2:**
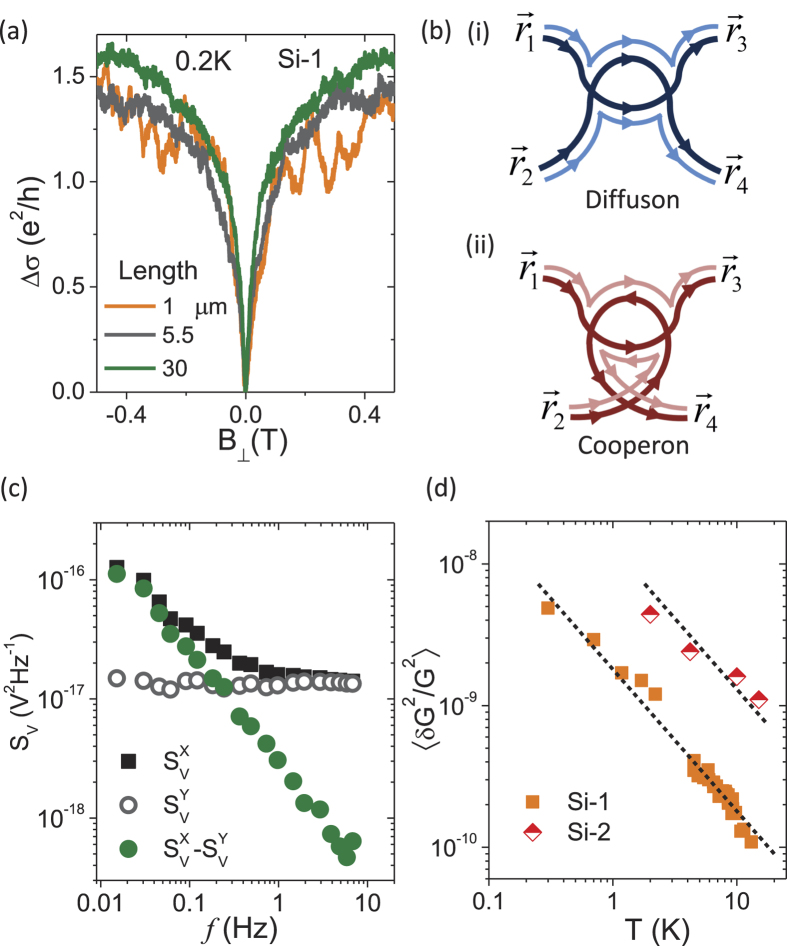
Estimation of UCF noise. (**a**) The magnetoconductivity, 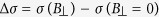, in a perpendicular magnetic field 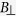 for highly doped Si:P delta layer, Si-1, having different distances between the voltage probes at 0.2 K. (**b**) Schematic representation of quantum propagators, Diffuson and Cooperon. (**c**) A typical 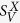, 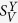 and 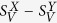, where 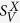 and 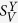 are the power spectral density of X-component and Y-component of voltage fluctuations respectively, for one of the Ge:P device. (d) The normalized variance of conductance fluctuations 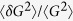 as a function of temperature for two Si:P devices, Si-1 and Si-2.

The UCF is manifested as aperiodic reproducible fluctuations (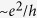) in conductance as a function of 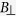, called *magneto-fingerprinting*, when the sample dimensions are of the order of phase coherence length 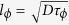. The *magneto-fingerprinting* has been observed at 0.2 K for device Si-1 of width 2 μm when the length of the *δ*-layer between the voltage probes is 1 μm as shown by the orange trace in Fig. 2a. As the length increases, the fluctuations decrease due to ensemble averaging and for the largest dimension, 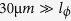 the conductance fluctuations are 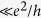 (olive green trace in Fig. 2a). To study the dependence of UCF on external parameters like magnetic field and temperature, we have measured the low frequency conductance fluctuations, which has been previously used to probe UCF and metal-to-insulator transition in bulk doped silicon^33^,^34^,^35^. The slow time-dependent fluctuations in conductance are recorded, which because ergodicity is analogous to ensemble fluctuations^23^,^33^,^36^,^37^,^38^. We employ an AC four probe balanced bridge technique to measure the time-dependent conductance fluctuations which is then digitally processed to obtain the power spectral density, PSD (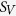), by using a fast fourier transform technique. The PSD of the out-of-phase component of voltage fluctuations is the background Johnson’s noise along with amplifier noise (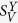) which is subtracted from the total noise (PSD of the in-phase component, 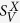) to obtain the noise from the sample (Fig. 2c). The details of the process can be found elsewhere^39^,^40^,^41^. From the measured 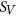, and known current *I* and voltage *V*, 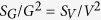 is computed. The normalized variance of conductance fluctuations can be calculated as, 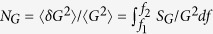, where 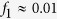 Hz and 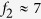 Hz define the experimental bandwidth. Using this procedure we have estimated the magnitude of UCF at different temperatures and 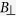.

As a function of temperature, 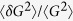 increases as the temperature reduces (for 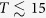 K) as shown in Fig. 2d for Si-1 (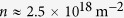) and Si-2 (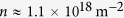) and is associated with quantum interference effects. The noise in this region is due to UCF which increases at low temperatures as 1/T as shown by the dotted black lines in Fig. 2d. The 1/T dependence can be understood from the Feng-Lee-Stone mechanism of conductivity fluctuations due to the movement of a single impurity atom. In this mechanism, the conductance fluctuations within a phase coherent box 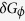 is^37^,^38^


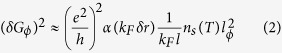


where 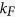, *l* and *n*_*s*_ are the Fermi wavevector, mean free path and the density of active scatterers respectively. The function *α*(*x*) represents the phase change of the electron wavefunction due to scattering off the moving impurity at a distance *δr*. It is important to mention that for these devices 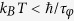 for the entire range of temperature and hence Eq. 2 is applicable. As 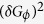 from different boxes are statistically independent of each other, the conductance fluctuations of the entire sample, 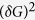, is related to 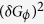 as,


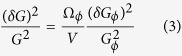


where 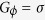 is the conductance of a single phase coherent box, 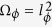 is the volume of one phase coherent box (or area for 2D systems) and *V* is the volume of the sample (or area 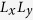 in the 2D case with 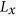 and 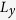 being the sample dimensions in the x and y direction respectively). Substituting for 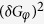 using Eq. 2, we get


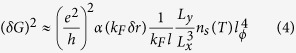


From Eq. 4 we get 
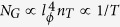
, where 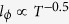 (as obtained from WL fits to the magnetoconductivity shown in Fig. 1 and Ref. 42) and *n*_*T*_(∝*T*) is the density of active two level fluctuators^37^,^38^,^42^. The observation of *magneto-fingerprinting* in 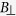 and the temperature dependence of 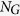 establishes the dominance of UCF in our devices.

### Scattering rates relevant to UCF from the B-dependent UCF

**Figure 3:**
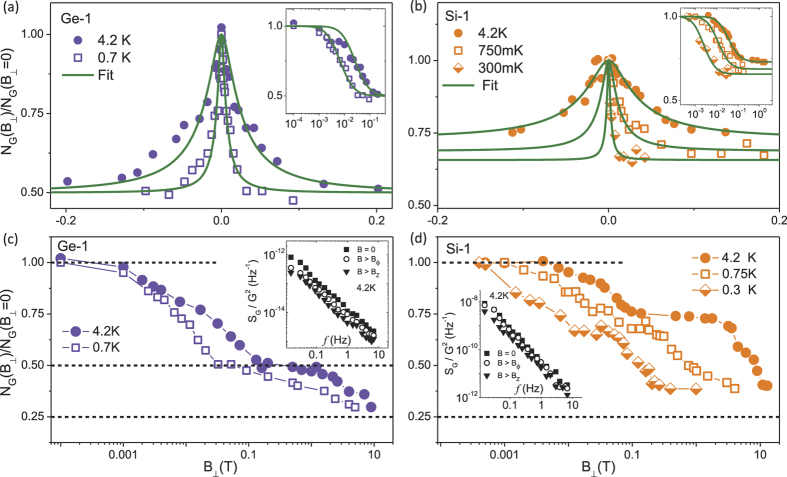
UCF magnitude in a perpendicular magnetic field. (**a**) 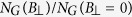 as a function of perpendicular magnetic field 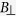 for device Ge-1 (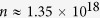 m^−2^) at 4.2 K (filled circles) and 0.7 K (open squares), where 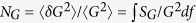 is the normalized variance of conductance fluctuations. The right inset shows 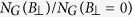 as function of 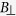 in the semi-log plot. The solid olive colored lines are fits to Eq. 6 of the main text. (**b**) 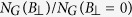 as a function of perpendicular magnetic field 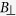 for device Si-1 (*n* ≈ 2.5 × 10^18^ m^−2^) at 4.2 K (filled circles), 0.7 K (open squares) and 0.3 K (half-filled rhombuses). The right inset shows 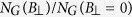 as function of 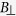 in the semi-log plot. The solid olive colored lines are fits to Eq. 7 of the main text. (**c**) 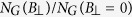 for Ge-1 at 4.2 K (filled circles) and 0.7 K (open squares) as a function of 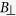 ranging from 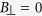 to 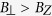, where 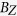 is the Zeeman field. The inset shows the power spectral density 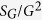 for 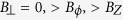. (d) 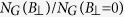 for Si-1 at 4.2 K (filled circles), 0.7 K (open squares) and 0.3 K (half-filled rhombuses) as a function of 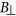 ranging from 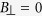 to 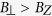, where 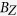 is the Zeeman field. The inset shows the power spectral density 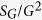 for 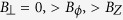. The dashed lines are put to indicate the reduction factors in a perpendicular magnetic field.

To determine the dephasing rate relevant to UCF, we have measured the 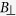 dependence of 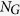 for devices of varying carrier density (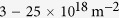) at different temperatures (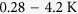). The UCF magnitude reduces by a factor of two at 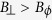, where 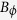 is the phase breaking field which introduces one flux quantum within a phase coherent box, as shown in Fig. 3a for the the highly doped Ge-1 *δ*-layer at 0.7 K (open squares) and 4.2 K (filled circles). A reduction in noise magnitude by a factor of two at the scale of 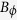 is expected for low-dimensional systems at low temperatures in the quantum coherent regime where the low frequency noise arises due to UCF^36^. A perpendicular magnetic field removes the Cooperons’ (self-intersecting trajectories, schematic in Fig. 2b) contribution to noise thus suppressing its magnitude by a factor of two as has been observed for various systems like metal films^37^, mesoscopic wires^43^, bulk Si^33^ and graphene^44^. Similarly for Si-1 *δ*-layer, the *N*_*G*_ reduces as 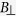 increases beyond 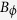 as shown in Fig. 3b at 0.3 K (half-filled rhombus), 0.75 K (open squares) and 4.2 K (filled circles). However, we do not observe a full factor of two reduction in *N*_*G*_ for Si-1 (it reduces to about 60–70% of the value at 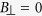). This happens due to a spontaneous breakdown of time reversal symmetry when the effective on-cite Coulomb interactions increase as has been elaborated in an earlier publication^42^. The 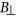-dependence of *N*_*G*_ can be fitted with the theoretical crossover function 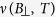 to estimate the scattering rates relevant to UCF. The exact form of 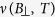 depends on whether the UCF is saturated or unsaturated. The measured UCF in a sample is said to be in the saturated regime if the time-dependent noise integrated over 20 decades of frequency equals the magnitude of magnetic field dependent UCF^28^,^37^. To confirm whether the Si:P and the Ge:P *δ*-layers are in the saturated or unsaturated regime we follow the method outlined in ref. 37. The measured time-dependent noise is related to the conductance fluctuations as^37^


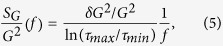


where, 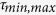 denotes the relaxation time of noise causing processes. From the magneto-fingerprinting data (Fig. 2a), we find that 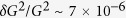, while from the power spectrum of the time-dependent noise we get, 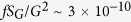. Substituting these values in the Eq. 5, we get, 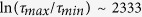. This suggests that the time-dependent noise has to be integrated over 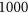 decades of frequency to saturate the UCF. Hence we can safely assume that the Si:P and Ge:P *δ*-layers are in the unsaturated regime.

The 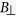-dependence of *N*_*G*_ (Fig. 3) was fitted with the crossover function 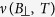, appropriate for unsaturated UCF, given by^36^


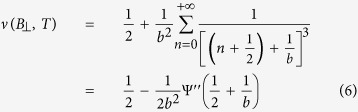


where 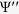 is the second derivative of the digamma function and 
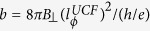
 with 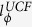 being the phase breaking length relevant to UCF. For devices where we do not observe the full factor of two reduction at 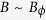, we have fitted the experimental data with the modified crossover function 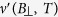 given by refs 43, 25, 27,


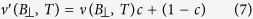


where *c* is the fractional UCF noise. The solid olive lines in Fig. 3a and b show fits to Eq. 6 and Eq. 7 respectively with 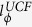 as the fitting parameter. The right inset of Fig. 3a and b shows the same plot and fits in the logarithmic scale. Using this procedure we have calculated 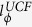 for all devices at different temperatures. The corresponding phase breaking time 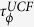 can then be calculated using the relation 
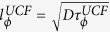
. As 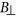 increases further (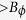), *N*_*G*_ reduces by an additional factor of two at 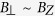 (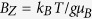 is the Zeeman field) due to the removal of spin degeneracy^43^ as shown in Fig. 3c and d for Ge-1 and Si-1 respectively (the corresponding PSD 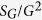 at 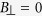 (filled squares), 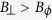 (open circles) and 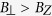 (filled triangles) for Ge-1 and Si-1 are shown in the inset in Fig. 3c and d respectively). Since the characteristic field scales 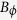 and 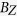 decreases with decreasing *T*, the reductions in *N*_*G*_ occur at a lower value of 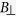 for lower *T* as shown in Fig. 3a,b,c and 3d. The observation that *N*_*G*_ reduces by factors of two at 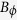 and 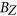 further confirms UCF as a major source of noise in these systems at low temperatures.

### Comparison of phase breaking rates relevant to WL and UCF

**Figure 4:**
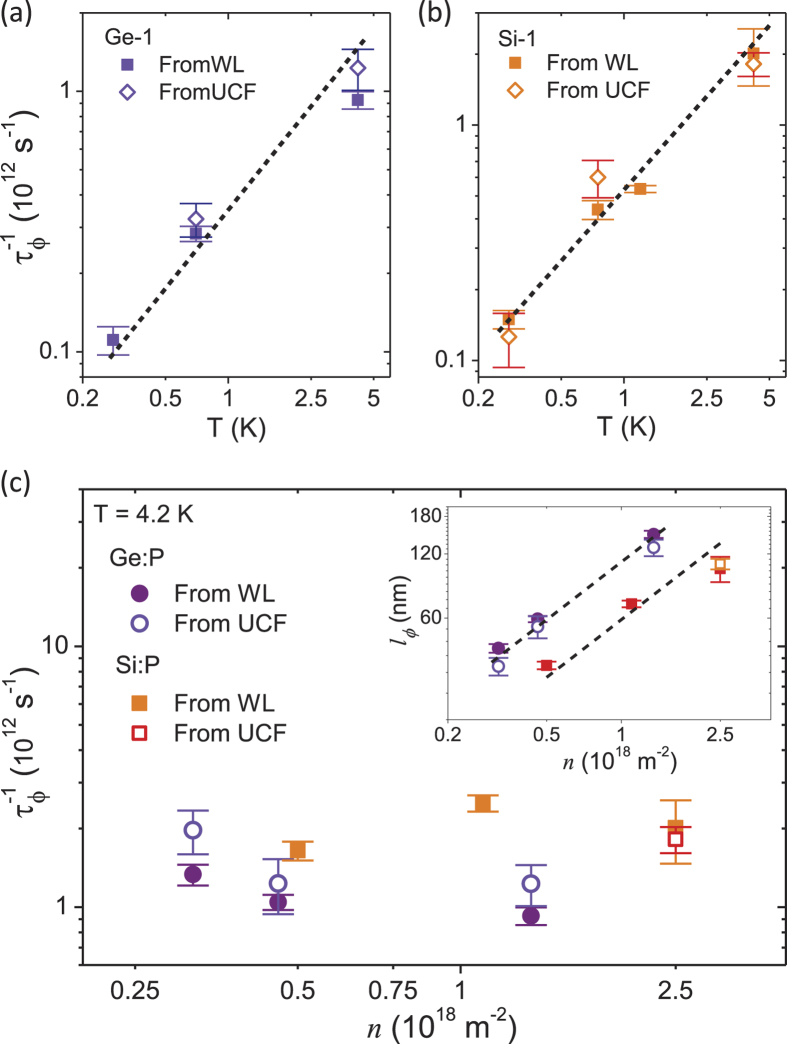
Comparison of dephasing rate relevant to WL and UCF. (**a**) Comparison of phase breaking rate 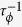 extracted from fits to the weak localization (WL) theory (filled symbols) and universal conductance fluctuations (UCF) theory (open symbols) at different temperatures for Ge-1. The dotted black line shows that 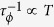. (**b**) Comparison of phase breaking rate 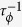 extracted from fits to the WL theory (filled symbols) and UCF theory (open symbols) at different temperatures for Si-1. The dotted black line shows that 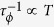. (**c**) Comparison of phase breaking rate 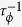 extracted from fits to the WL theory (filled symbols) and UCF theory (open symbols) for Si:P and Ge:P devices of varying density at 4.2 K. The inset shows the comparison of phase breaking length relevant for WL and UCF for Si:P and Ge:P devices of varying density at 4.2 K. The dotted black lines show that 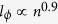.

The comparison between phase breaking rate 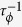 for WL and UCF, as extracted from theoretical fits to magnetoconductivity and *N*_*G*_ vs 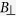 respectively, is illustrated in Fig. 4 for different temperatures and density. The magnitude of 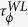 and 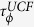 agree well with each other at all temperatures for Ge-1 and Si-1 as shown in Fig. 4a and b respectively. The corresponding scattering rates 
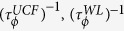
 are 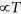 (dotted lines in Fig. 4a and b) indicating that the dephasing is due to electron-electron scattering (or Nyquist dephasing)^11^.

The equivalence of 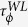 and 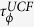 is illustrated for devices of varying density in Fig. 4c. For Ge:P 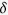-layers the comparison between the two scattering times have been made in the density range 0.32–1.35 × 10^18^ m^−2^. However for the Si:P 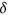-layer, we were not able to extract 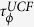 for lower density devices since the UCF noise was suppressed at 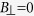 due to a spontaneous breakdown of time reversal symmetry in the strongly interacting regime as has been explained in Ref. 42. The inset of Fig. 4c shows that 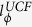 and 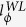 (phase breaking length corresponding to UCF and WL respectively) agree with each other for all densities investigated in our experiment. The dotted black line shows that 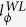 (or 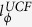) ∝*n*^0.9^. Table 1 shows the values of 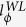 and 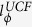 for devices of varying density at different temperatures. The ratio 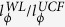 ranges from ~0.95–1.2 implying a maximum difference of ~20% between the two values which is within the limits of statistical uncertainty.

## Conclusion

In conclusion, we have studied the quantum interference phenomena in 2D Si:P and Ge:P *δ*-layers. Weak localization corrections to conductivity and universal conductance fluctuations have been measured as a function of temperature and perpendicular magnetic field. The phase breaking rate 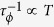, which indicates that electron-electron scattering is the dominant dephasing mechanism in these systems. The relevant dephasing rate (and hence the phase coherence length) corresponding to WL and UCF are in quantitative agreement with each other for devices of different doping densities indicating that the same scattering times govern WL and UCF for dephasing due to electron-electron interaction. The result is significant as WL and UCF are fundamental concepts in solid state physics and an equivalence of the corresponding scattering rates reiterates that the two phenomena are analogous even when the system is purely two dimensional in nature.

## Additional Information

**How to cite this article:** Shamim, S. *et al*. Dephasing rates for weak localization and universal conductance fluctuations in two dimensional Si:P and Ge:P *δ*-layers. *Sci. Rep.* **7**, 46670; doi: 10.1038/srep46670 (2017).

**Publisher's note:** Springer Nature remains neutral with regard to jurisdictional claims in published maps and institutional affiliations.

## Figures and Tables

**Table 1:** The phase coherence lengths 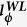 and 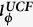 extracted from weak localization fits to magnetoconductivity in a perpendicular magnetic field (*B*_⊥_) and theoretical crossover function fits to *B*_⊥_ dependence of conductance fluctuations, respectively for various Si:P and Ge:P *δ*-layers at different temperatures T. *n* is the carrier density as determined from Hall measurements.

Sample	*n* (10^18^ m^−2^)	*T* (K)	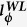 (nm)	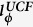 (nm)	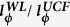
Si-1	2.5	4.2	102	108	0.95
		0.75	220	188	1.17
		0.28	376	410	0.92
Ge-1	1.35	4.2	148	129	1.15
		0.7	268	251	1.07
Ge-2	0.46	4.2	60	55	1.1
Ge-3	0.32	4.2	43	36	1.2
